# Approach-avoidance orientations can predict young children’s decision-making

**DOI:** 10.1371/journal.pone.0288799

**Published:** 2023-07-24

**Authors:** Avi Benozio, Reshit Cohenian, Robert Hepach

**Affiliations:** 1 Hebrew University of Jerusalem, Jerusalem, Israel; 2 Oxford University, Oxford, United Kingdom; Universitat Jaume I Departament d’Economia, SPAIN

## Abstract

When facing situations that involve risk and reward, some may focus on the opportunity for reward, whereas others may focus on potential risks. Here, we used an original set of pictorial scenarios to try and predict 3- to 8-year-olds’ reward-seeking and risk-avoiding behavior in three decision-making scenarios (*N* = 99; *M*_age_ = 5.6; 47% girls). We found that children’s reward-risk tendencies did not predict *sharing* behavior in a dictator-game ‘sharing’ task. However, they predicted children’s *monopolizing* behavior in a dictator-game ‘taking’ task and their preferences between taking home a ‘risky’ or a ‘safe’ reward in a novel prize-preference task. Overall, using a set of original pictorial scenarios to assess individual differences early on in development now provides initial evidence that bridges individual differences and decision-making domains and exposes behavioral patterns that were thus far hidden.

## Introduction

Any adaptive species must somehow balance between avoiding potential threats (e.g., predators), and obtaining potential benefits (e.g., prey). In humans, this basic psychological tension is broadly reflected in two independent neural substrates representing intrinsic motivations: Behavioral Inhibition Systems (BIS) and Behavioral Approach Systems (BAS). BIS is more sensitive to cues of punishment and loss, thus regulating avoidant behavior, while BAS is more susceptible to signals of reward, thus facilitating appetitive behavior [1–8].

One standard assessment of BIS-BAS orientation in adulthood is with a self-report questionnaire [8], which exposed meaningful correlates with frontal EEG asymmetry. Specifically, avoidance-oriented emotional responses (i.e., negative affect in general) are linked with right frontal regions (i.e., BIS), whereas approach-oriented responses (i.e., positive affect) are linked with left frontal areas (i.e., BAS) [5]. Broadly speaking, individuals with an approach motivational orientation may be more likely to engage in social activities, take risks, and express positive emotions. Complementary, individuals with an avoidant motivational orientation may be more likely to shy away from new social interactions, avert risks, and express negative emotions [5, 9–12]. Notably, an age-downward version of the adult questionnaire was designed for young adolescents (8-12-year-olds) [13], and although did not converge to the full factorial structure of the original questionnaire, it was predictive of personality traits and symptoms of psychopathology. For example, BIS was correlated with higher levels of internalizing symptoms (e.g., Anxiety and Depression), whereas BAS was correlated with externalizing ones (e.g., Hyperactivity and Aggression).

The theoretical and pragmatic interplay between approach, avoidance, and inhibitory capacities provides significant associations between temperament in infancy and the developed adult personality [7, 14, 15], and “motivational imbalance” in early childhood correlates with maladaptive behavior in the social sphere [2, 16]. Several comprehensive models suggest that one’s motivational orientation (i.e., BIS or BAS) should be particularly predictive in unfamiliar or ambiguous contexts which involve opportunities for risk and reward [2, 15]. However, unlike in adults or adolescents, measuring BIS-BAS orientation in early childhood is still an open challenge [2].

This challenge is typically addressed by resorting to other measures or paradigms, such as asking caregivers to report about their children [17–21], observational paradigms [22], ‘game-like’ tasks [23], or longitudinal studies [14, 24, 25]. Such efforts reveal intriguing insights regarding continuity and change in motivational orientation throughout the lifespan [2, 14, 26], yet each also bears inherent disadvantages. Specifically, caregivers’ reports provide an *indirect* subjective assessment, observational methods demand particular *expertise*, and ‘game-like’ tasks focus on *specific* cognitive capabilities such as inhibitory control, working memory, and attention rather than the broad BIS-BAS theoretical framework, and longitudinal studies are *expensive* and thus rare.

Moreover, regarding the developmental trajectory of risk-taking behavior, a recent meta-analyses [27] showed that in laboratory tasks children and young adolescents (5–10 and 11–13 years old, respectively) are equally high at risk-taking, compared to adolescents and adults (14–19 and 20–65 years old, respectively). Thus, crafting a new tool for assessing BIS-BAS orientation will not only provide insights into a developmental period in which such assessments are lacking but will also address a period in which risky behavior is high.

The earliest efforts to assess temperament were carried out by Garcia-Coll et al. [25] and Kagan et al. [28] who introduced two-year-olds to unfamiliar scenarios, such as interactions with strangers, new environments, and unpredictable toys. Based on behavioral and psychophysiological assessments, toddlers were categorized into one of three groups: inhibited, uninhibited, or neither. Subsequent research that followed these innovative studies, further revealed that having a history of behavioral inhibition in childhood predicts an attentional bias toward potential threats in adolescents [29], and when this heightened attentional bias is accompanied by heightened negative self-evaluation, then the likelihood of developing social withdrawal in later adulthood increases [14, 30, 31]. Notably, associations between temperament in childhood and adaptive behavior in adulthood cannot be separated from the reciprocal feedback from the immediate social surroundings (e.g., parental encouragement of certain temperamental styles) [32] as well as the broader socio-cultural one (e.g., cultural differences in valuing and promoting different temperamental styles) [33, 34].

In addition to the association between temperament and risk, another related aspect would be gender. First, regarding temperament, one meta-analysis which focused on gender differences in temperament from infancy to 13-years-of-age [35], reports large differences favoring girls in aspects related to ‘effortful control’ (e.g., attention focus, shifting, inhibition, and sensitivity), and medium differences favoring boys in aspects related to ‘surgency’ (e.g., activity, approach, pleasure intensity, impulsivity). Second, regarding risk-taking behavior, women have been largely found to be more averse to risk than men in economic-like laboratory tasks [36], and similar findings were found in young children in observational and laboratory settings [37, 38], across several cultures [39], and particularly in competitive dyadic contexts [40, 41].

Together, assuming that one’s biological, parental, cultural, and personal history converge to manifest in a dominant motivation to either approach potential benefits OR to avoid potential risks, then BIS-BAS orientation in early childhood should predict actual behavior. To address this challenge, we created a child-friendly adaptation of the widely used BIS-BAS questionnaire [8]. The BIS-BAS-Book (here *BBB*) comprises a series of original pictorial scenarios that present a protagonist facing two possible courses of action—risky & rewarding or conservative & safe. With the BBB, children choose the course of action they think a protagonist should take, and a BIS-BAS orientation score is computed. The rationale, as alluded to before, is that especially in unfamiliar scenarios in which children have little prior experience, BAS-oriented children will tend to pursue potential self-benefits, whereas BIS-oriented children will tend to avoid potential risks.

The power of the BBB to predict children’s actual behavior was assessed in three distributive decision-making games that vary in their degree of familiarity to the child. The first decision-making scenario is a resource distribution task known as the Dictator game [42, 43]. In this game, one individual (i.e., the ‘dictator’) is allotted a divisible endowment (e.g., money for adults, or stickers for children), and can share with a second individual as he/she sees fit. The recipient receives whatever the ‘dictator’ dictated, and the two parties depart. With this simple game, adults from WEIRD and non-WEIRD societies typically share about 30% of their endowment [44], which is far from the canonical model of purely selfish agents (i.e., who should share nothing). In development, empirical findings thus far have shown that children gradually shift from acting primarily upon selfish considerations at around 3 years of age (e.g., 60% share ‘nothing’ and 10% share ‘half’) to acting upon equity-based norms from about 6 years of age (e.g., 20% share ‘nothing’ and 35% share ‘half’) [45, 46]. However, there is also considerable heterogeneity within each age cohort due to a series of factors, such as the personal characteristics of the ‘dictator’ (e.g. socio-economic status) [47, 48], attributes of the recipients (e.g. being ‘needy’ or not) [49], the relations between the participants (e.g. group membership) [50], or attributes of the endowment itself (e.g. attractiveness) [46, 51].

Dictator-game ‘sharing’ could be considered a relatively familiar event (e.g., “*sharing is caring”*, as often stated and encouraged in kindergartens), in which children across cultures start similarly, and share about 25–30% of their endowment [52]. Thus, the main dilemma in this game would be how many resources to forgo for the sake of another, and children will likely manifest whatever habituated behavior they already have (e.g. generous, egalitarian, or selfish) regardless of their BIS-BAS motivational orientations. Alternatively, if a ‘sharing’ scenario is unfamiliar to young children, then we would suspect that BAS-oriented children, who are arguably more focused on obtaining rewards, will share less with others.

The second decision-making game is a variation of the dictator game noted above and was first introduced by Eichenberger and Oberholzer-Gee [53–55]. In this game, one can decide how many resources to *take* from another (i.e., dictator-game ‘taking’). The ‘sharing’ and ‘taking’ scenarios may seem complementary, but recent findings with adults emphasize a general aversion to ‘taking’ from others, and that such a ‘taking aversion’ may be somewhat stronger among women [56, 57]. To our knowledge, the early emergence of ‘taking aversion’ was never examined in early childhood, though limited evidence does suggest that 3-6-year-olds appreciate more puppets who ‘shared’ resources with them than puppets who ‘took’ resources from them, even if the final number of rewards at the children’s possession was identical, suggesting that at 3-years-of-age, children distinguish between social signals such as ‘sharing with’ from ‘taking from’ [58]. Unlike the ‘sharing’ scenario, the ‘taking’ scenario is supposedly less common in children’s daily life, and taking resources from others is normatively prohibited. Thus, if a ‘taking’ scenario is relatively unfamiliar, and pits conflicting motivations such as the desire to obtain benefits on the one hand, and an aversion to ‘taking from others’ on the other, then we would expect BAS-oriented children to take *more* from others (i.e., being focused on self-benefits) and expect BIS-oriented children to take *less* (i.e., being focused on the aversion from ‘taking’).

In the ‘sharing’ and ‘taking’ dictator games, which are dyadic tasks in nature, there could be some underlying expectation of other-regarding considerations or social norms [59]. Thus, the third and last decision-making scenario was designed to remove such potential confounds. In this third scenario, a novel prize-preference task was used, offering children to pick one reward (out of many) to take home with them. Previous research with children demonstrated that various cues could steer their preferences for some objects over others. For instance, the associations between an object and social categories (e.g., age, gender) [60], having information about the preferences of other children [61, 62], and even the scarcity or abundance of objects [63, 64]. Here, we created an ecological scenario wherein children are given various prizes to choose from but can pick only **one** to take home with them. Notably, while children could see the content of most prizes (i.e., thus considered being ‘safe’ choices), one prize had an opaque wrapping (i.e., representing a ‘risky’ choice due to its unknown content). In this scenario, BAS-oriented children are expected to choose a ‘risky’ prize, whereas BIS-oriented children are expected to choose one of the ‘safe’ prizes to take home with them.

In sum, the current research aims to assess the power of the BIS-BAS-Book (i.e., BBB) to predict children’s behavior in three decision-making scenarios. Since approach and avoid orientations are most predictive and relevant in unfamiliar or ambiguous situations [2, 15], our first hypothesis is that BIS-BAS orientation will be more predictive in unfamiliar scenarios (i.e., dictator-game ‘taking’ and prize-preference tasks) than in the familiar one (i.e., dictator-game ‘sharing’ task). A second, more specific hypotheses are that BAS-oriented children will likely take more resources in the *‘taking’* scenario than BIS-oriented children, and will be more likely to take home with them a ‘risky’ prize in the prize-preference task. Notably, we did not have an apriori hypothesis about gender since it may be subsumed under children’s BIS-BAS orientation. Moreover, since the ratio of BIS vs. BAS-oriented children was previously found stable across ages, we did not expect age related differences in BIS-BAS orientation scores [25, 26]. If successful, the BBB will help bridge two relatively distinct research domains, namely, the study of individual differences and decision-making across contexts.

## Methods

### Participants

99 3-8-year-olds (*N* = 99, *M*_age_ = 5.6, *SD* = 1.3 years; 47 girls) were recruited from the Bloomfield Science Museum in Jerusalem, Israel. Participants were from mixed SES backgrounds, and all had signed parental permission to participate (research ethics committee #1281119). Sixteen additional children were tested but excluded due to experimenter errors. The G*Power software package (Ver 3.1.9) was used to conduct a power analysis to estimate the number of participants required for a regression model with four independent variables (BBB-orientation score, age, gender, and caregiver’s BIS-BAS-orientation score). Detecting a medium effect size of f^2^ = .15 at 80% power requires 85 participants. Our sample was ~15% larger.

### Design

The design consisted of three sequential phases: **(1)** The *BBB phase*, in which all children completed the BBB that yielded an individual raw BIS-BAS orientation score (see S1 Table and S2-S6 Figs in S1 File). **(2)** A *Dictator-game phase*, in which participants were randomly assigned to one of two dictator-games, that is, a dictator-game ‘*sharing*’ condition (n = 49; M_age_ = 5.6; SD = 1.5; 43% girls) OR a dictator-game *‘taking’* condition (n = 50; M_age_ = 5.7; SD = 1.2; 52% girls). **(3)** A *Prize-preferences phase* in which all children participated in the prize-preference task and chose one reward out of several to take home with them.

### Materials

#### The BBB phase

**Fig 1:**
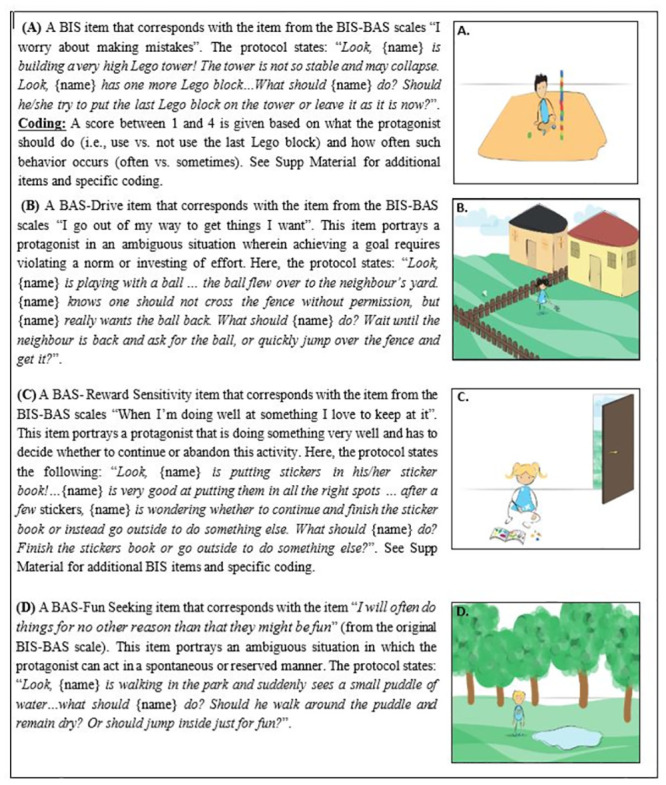
Four exemplars (out of 18) for the BBB pictorial scenarios. Participants receive a score (1–4) for each of the BIS and BAS scales. See S1 Table and S2-S6 Figs in S1 File for full list of items and specific coding.

Content: Based on the original BIS-BAS scales [8], and the simplified version for 8-12-year-olds [13, 65], we have simplified the original scales to address 3 to 8-year-olds in the following manner: First, we have shortened the questionnaire to 12 items (instead of 24) (see S1 Table in S1 File). Second, we used original illustrations depicting scenarios common in children’s daily lives. In each scenario, a protagonist encountered a dilemma, and participants were asked to choose between two behavioral options (Fig 1 and S2-S6 Figs in S1 File).

Protagonists: Protagonists’ characteristics, such as **age** and **name**, were identical to participants’ (i.e., explicitly stated by the Experimenter), and protagonists’ physical characteristics, such as **gender** and **hair color**, were also matched by using an appropriate set of illustrations. We did this to increase the likelihood that participants will provide their own tendencies rather than normative statements (as in 3^rd^-party judgment).

Order and coding of items: We created 16 books (i.e., four different orders of items X four protagonists who vary in gender and hair color). Each item in the BBB was scored on a 1–4 point scale to match the coding in the original BIS-BAS scales for adults (i.e., a 1-4-point scale from “very false to me” to “very true for me”).

BIS-BAS Orientation Score: We based our BIS-BAS-orientation score on prior research that utilized an approach-avoidance asymmetry score [5, 66, 67]. Various methods can be employed to analyze BIS and BAS motivations [8, 21, 65, 68], but we used the BIS-BAS asymmetry method due to its close tie to the concept of having a dominant motivational orientation. To calculate the score, we acquired Z-BAS and Z-BIS scores (applying a z-transformation) upon completion of the sample and then calculated the individual’s BIS-BAS orientation score (i.e., |Z-BAS|—|Z-BIS|). Higher scores represent higher BAS orientation, whereas lower scores represent higher BIS orientation. In addition, the caregivers of the participants filled out the original adult version of the BIS-BAS scales questionnaire about their child to obtain an indirect assessment.

#### The dictator-game phase: Sharing and taking

We employed ten identical erasers as resources for distribution. Each eraser was wrapped with a different color to maintain the children’s interest and participation throughout the games. In the ‘sharing’ game, the children were presented with a video of an unfamiliar, gender- and age-matched peer (see S7 Fig in S1 File) and asked to decide how many rewards they wished to share with him/her. Similarly, in the ‘taking’ game, children were asked to decide how many rewards they would like to take from a peer.

#### The prize-preferences phase

**Fig 2:**
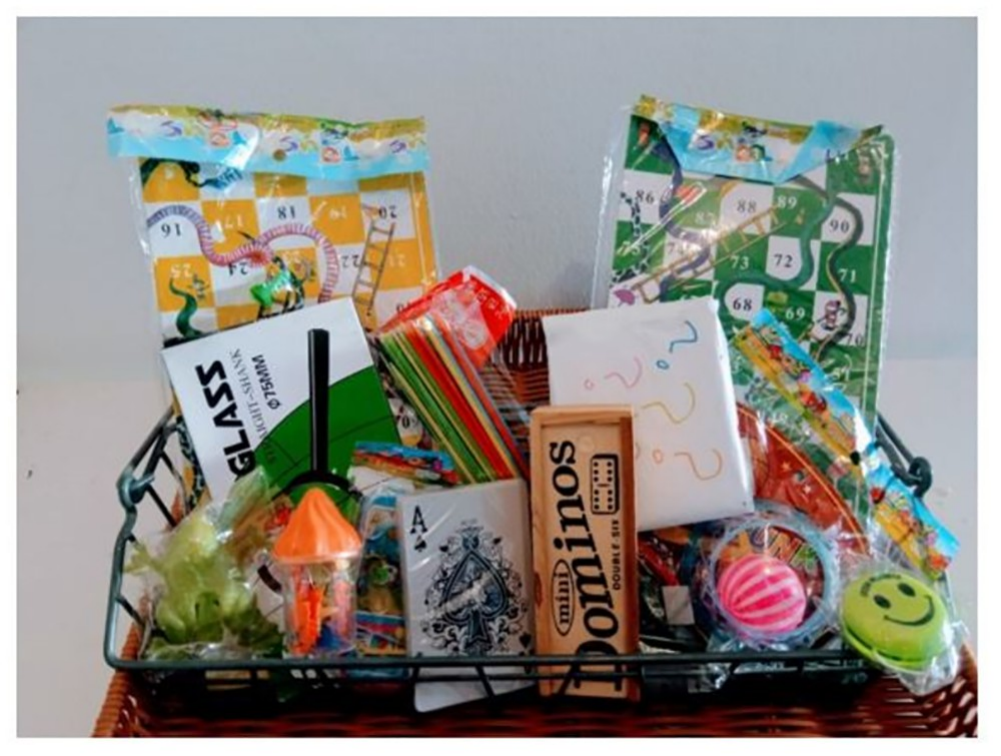
Prizes in the prize-preference task. Children were free to choose **one** prize to take home with them. 11 prizes were visible to children (i.e., ‘*safe*’ choices), and one was not (i.e., a ‘*risky*’ choice). The position of the ‘risky’ prize was counterbalanced (left, middle, right).

An experimenter presented participants with a basket containing 12 prizes, 11 of which were visible (i.e., representing ‘safe’ choices) and one wrapped in white paper with question marks all over (i.e., representing a ‘risky’ choice). The prizes were part of what is regularly given to children as compensation for participating in studies in the Museum (Fig 2). Presenting a basket with several ‘visible’ prizes ensured that every child could find an attractive ‘safe’ prize regardless of age, gender, and background and would not be ‘nudged’ towards the ‘risky’ option due to a lack of a suitable satisfying alternative.

### Procedure

#### BBB phase

Children sat in a designated quiet zone in the museum, and caregivers waited outside during the procedure while filling out a BIS-BAS questionnaire about their child. The Experimenter (E1) presented the BBB with the appropriate protagonist (i.e., hair color, gender, age, name), and children’s verbal answers were audio-recorded for later coding.

#### Dictator-game phase

Upon completing the BBB phase, E1 stated, “*OK*, *now that we have finished the book*, *I want to show you a game … in this game*, *you can decide how to distribute prizes between you and another child*”. E1 opened a laptop and presented a video of gender and age-matched unfamiliar peer. In the ‘sharing’ scenario, E1 placed ten rewards closer to the participant, asked participants to count the rewards, and stated, “*Now*, *you can decide how many rewards to give to* {peer’s name}”. In the ‘taking’ scenario, E1 placed ten rewards closer to the peer (i.e., closer to the laptop screen), asked participants to count them, and then stated, “*Now*, *you can decide how many rewards to take from* {peer’s name}.”

#### Prize-preferences phase

Upon completing the dictator-game phase, E1 stated, “*Great*, *now that you have completed the game*, *you deserve a real prize*!”. A second experimenter (E2) joined and invited the participant to come with her to choose a ‘real’ reward to take home. E2 guided the participant to a different zone in the museum and presented a basket with 12 prizes—11 were transparent, and 1 was wrapped in white paper with question marks. E2 was blind to the participant’s answers, assigned dictator-game condition, and distributive behavior in the previous phases (i.e., the BBB and Dictator games). E2 stated: “*So since you played so nice*, *you can choose whatever you wish to take home*”. E2 mentioned the names of the various prizes so all children would be aware of the variety (e.g., “*You can choose X*, *Y*, *Z*, *…*”), and when reached the ‘risky’ prize said, “*or this one*… *I don’t know what’s inside…*”

## Results

### The BBB phase

The ratio of BIS- or BAS-oriented individuals is stable per age cohort (but can change at the individual level) [25, 26]. To ensure this is the case, we divided the sample into three age groups (i.e., 3–5, 5–7, and 7-9-year-olds), and used a generalized linear model (GLM) with BIS-BAS orientation score as the dependent variable, and age group as a predictor. Likelihood ratio test comparison between the full and null models was non-significant (Χ^2^_(96)_ = 3.22, p>.4), suggesting no differences in BIS-BAS orientation scores between age groups. There ratio of BIS/BAS-oriented children within each age group (i.e., 3–5, 5–7, and 7-9-year-olds) was also similar and close to 1 (0.74, 1, and 0.77; respectively), suggesting a relatively equal number of BIS and BAS-orientated participants per age group. No correlations were found between the BIS-BAS orientation scores obtained via the BBB and caregiver’s reports (see §1.2 in S1 File). Such absence of correlations may not be surprising, given prior mismatches between the original scales used for adults and a simplified version for 8-year-olds [8, 13, 65]. This issue will be discussed further in our final discussion. In the sections to follow, we assess the predictive power of children’s BIS-BAS orientation on their actual behavior and use both scores obtained via the BBB and the caregiver’s reports, without the concern from multicollinearity.

### The dictator game phase

#### A ‘sharing’ scenario

**Fig 3:**
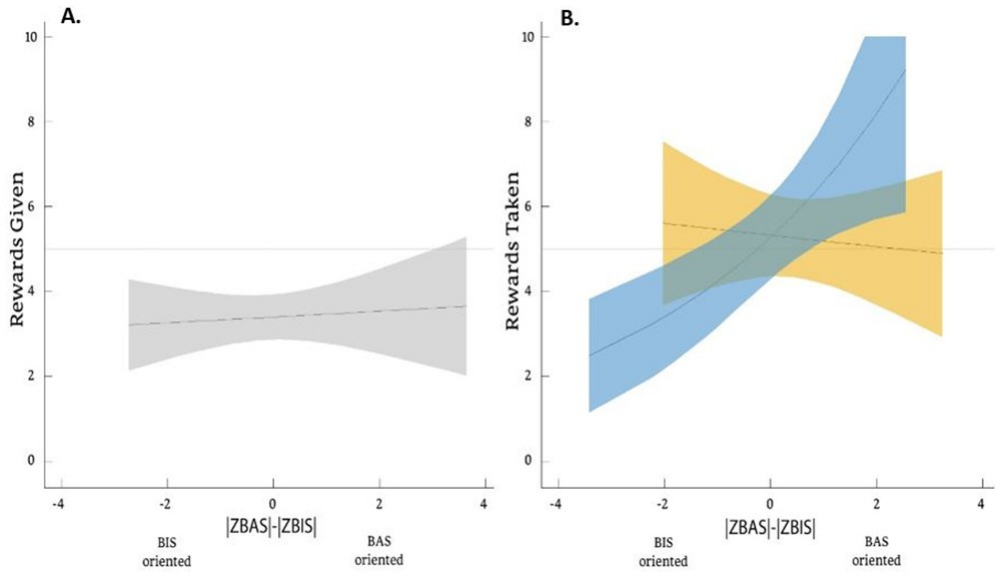
Children distributive behavior. In a ‘sharing’ scenario **(A)**, and a ‘taking’ scenario **(B)** as a function of their BIS-BAS orientation. Gray polygons represent all children, whereas blue polygons represent boys, and yellow polygons represent girls (95% CI).

A generalized linear model (GLM) included the number of rewards shared as a dependent variable. Independent variables were direct BIS-BAS orientation score (via the BBB), indirect BIS-BAS score (via the caregiver’s report), gender, all interactions between them, and age in months as a covariate. Likelihood ratio test comparison between the full and null model (which controlled for age and gender) was non-significant (*Χ*^2^_(40)_ = 3.66, *p* = .72), and children shared about a third of their endowment (*M* = 33.8%; *SD* = 24.2%) regardless of age, gender, or BIS-BAS orientation scores (Fig 3A).

#### A ‘taking’ scenario

We used an identical analysis (GLM), which included the number of stickers taken as the dependent variable, and as predictors—direct BIS-BAS orientation score, indirect BIS-BAS score, gender, all interactions, and age in months as a covariate. Likelihood-ratio-test comparison between the full and null model (which controlled for age and gender) was significant (*Χ*^2^_(41)_ = 12.61, *p* < .05), and yielded a two-way interaction between the direct BIS-BAS orientation score and gender (*X*^2^_(1)_ = 6.43, *p* = .01), and a main effect for the direct BIS-BAS orientation score (estimate ± *SE* = 0.09 ± 0.05, *z* = 2.06, *p* < .05). We followed the two-way interaction with two separate GLMs per gender. For boys: Likelihood ratio test comparison between the full model (including age and direct BIS-BAS orientation score) and null model was highly significant (*Χ*^2^_(21)_ = 11.22, p < 0.001), with a main effect for the direct BIS-BAS orientation score (estimate ± *SE* = 0.22 ± 0.06, *z* = 3.28, *p* = .001), suggesting that BAS-oriented boys took ~80% of others’ resources. Conversely, BIS-oriented boys took ~20% (Fig 3B). For girls: An identical analysis for girls yielded no significant difference between the full and null models (*Χ*^2^_(23)_ = 0.17, *p* = .68), suggesting that regardless of girls’ BIS-BAS orientation score, they took ~50% of other’s resources (*M* = 52.7%; *SD* = 30.1%).

Summary: Children’s sharing rates fit well with what is known from prior research on dictator games (e.g., 46). Whether assessed via the BBB or the caregivers, children’s BIS-BAS orientation did not predict their ‘sharing’ behavior. One explanation for the absence of findings in this regard would be that familiar contexts (e.g., sharing with others) do not elicit an ‘approach-avoid conflict’. Thus, habituated behavior can be expressed (i.e., generous, fair, or stingy) regardless of individual differences in risk-reward sensitivity. If true, we should expect a relation between BIS-BAS orientation and children’s behavior in a less familiar scenario, such as the dictator-game ‘taking’. Indeed, unlike the ‘sharing’ scenario, the BBB predicted children’s behavior in the ‘taking’ game. Importantly, without accounting for individual differences, we would wrongly conclude that all children took resources to reach an egalitarian outcome (i.e., 53.3%, 52.7%; boys and girls, respectively). However, a new picture emerged when including individual differences: BAS-oriented boys monopolize resources from others, whereas BIS-oriented boys are far less willing to do so. Conversely, girls presented a stable egalitarian tendency regardless of their motivational orientation. These findings echo the recent findings with adults by which women were found to have a stronger ‘taking aversion’ [56] but also reveal a similar tendency among BIS-oriented boys. Importantly, even though the reasons for gendered behavior in early childhood should be further explored, our findings here align with prior established gender differences in childhood, which revolve around social comparison and competitiveness [40]. Here, the BBB seems to at least identify those who were most competitive and/or less averse to monopolizing resources, i.e., BAS-oriented boys.

The ability to predict children’s behavior in the ‘taking’ scenario is aligned with the idea that a ‘taking’ scenario may present a more salient conflict than ‘sharing’, at least for boys, for which one’s motivational orientation strongly predicted behavior. One explanation for the inability to predict girls’ behavior could be that the BBB is somewhat biased. This explanation seems unlikely given that the protagonists were gender-matched, the pictorial scenarios included a wide range of daily events, and the range of girls’ BIS-BAS scores was similar to boys’ (Fig 3). A second explanation for girls’ egalitarian behavior would be independent of individual differences, but rather girls’ more general tendency towards egalitarianism [69]. It will be further discussed following the final decision-making task, which is the prize-preferences task.

#### The prize-preferences phase

**Fig 4:**
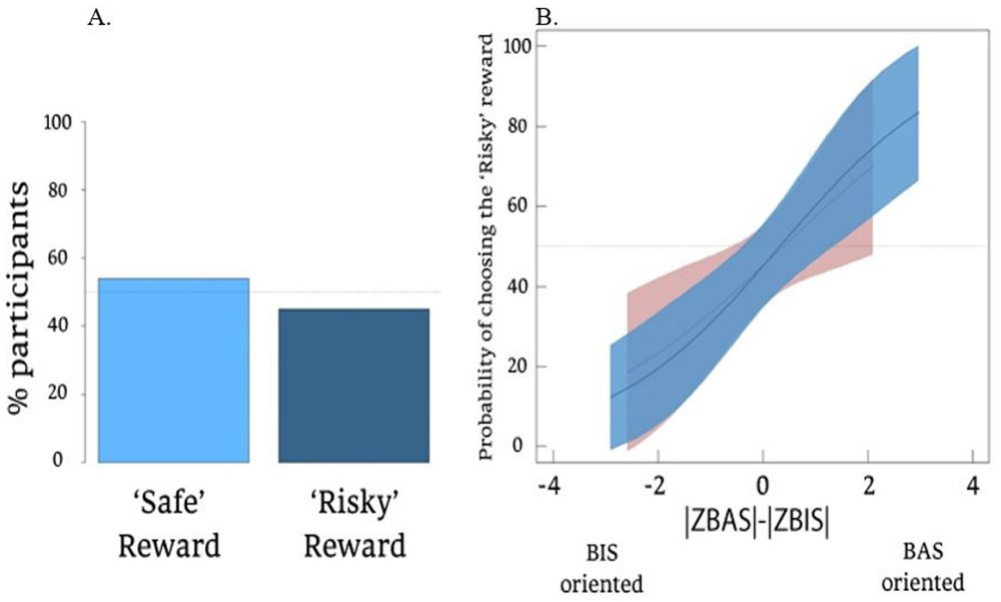
Choice preferences of ‘safe’ and ‘risky’ rewards. Without accounting for BIS-BAS orientations **(A)**, and with accounting for individual differences in BIS-BAS orientations **(B)** as obtained via the BBB (in blue) and caregiver reports (in red) (95% CI).

A chi-square test for children’s choices (‘safe’/‘risky’ rewards) was non-significant (*Χ*^2^_98_ = 54, *p* = .99), as 55% chose one of the visible, ‘safe’ rewards and 45% chose the unknown, ‘risky’ reward, suggesting no initial bias towards either type of reward (see Fig 4A). Second, a generalized linear model (GLM) included children’s choice (‘safe’/‘risky’) as the dependent variable, and as predictors—direct BIS-BAS orientation score (via the BBB), indirect BIS-BAS score (via the caregiver’s report), gender, all interactions between them, and age in months as a covariate.

Likelihood Ratio Test comparison between the full and null model (which controlled for age and gender) was significant (*Χ*^2^_(90)_ = 14.3, *p* < .05). Three and Two-way interactions were non-significant (all *p*s > .25), and only two main effects were found. The first was for the direct BIS-BAS orientation score (estimate ± *SE* = 0.44 ± 0.17, *z* = 2.62, *p* < .01), suggesting that BAS-oriented children are likely to choose a ‘risky’ reward above chance-level and BIS-oriented children are likely to select a ‘safe’ reward above chance level (Fig 4B). A second, weaker, main effect was for the indirect BIS-BAS score (estimate ± *SE* = 0.32 ± 0.15, *z* = 2.06, *p* < .05). This main effect followed the same pattern as the direct BIS-BAS orientation score but was predictive only for BIS-oriented children, who were likely to choose ‘safe’ rewards, above chance level.

Summary: We would mistakenly think that children do not distinguish between “safe” and “risky” choices in a simple prize preference task without accounting for individual variation in BIS-BAS orientation (i.e., 50–50; Fig 4A). However, the inclusion of individual differences exposed two distinct sub-groups, each having a significant and opposite preference—one that prefers to choose a ‘risky’ reward and a second that prefers a ‘safer’ option. Crucially, while the BBB was able to predict monopolizing behavior only among boys (i.e., in the dictator-game ‘taking’), here, both boys’ and girls’ prize choices were predicted, thus minimizing the concern of the BBB being biased. Instead, what may explain the differences in the ability to predict boys’ and girls’ behavior could be the difference between ‘social’ situations which affect others (e.g., ‘taking’ from others) and ‘non-social’ ones which affect only oneself (e.g., choosing a prize for self).

## General discussion

The idea of BIS-BAS orientations was initially inspired by animal models [6], influenced a host of psychological theories [70], and yielded a pragmatic, well-established adult questionnaire [8]. Here, the BBB was devised to provide initial evidence for individual differences in early and middle childhood [23, 71, 72]. Notably, the power of the direct and indirect BIS-BAS orientation scores to predict children’s behavior was aligned (i.e., BBB and parental reports)—specifically, in familiar dyadic interactions (i.e., ‘sharing’), both measures were *unsuccessful* in predicting children’s behavior. Then, only the BBB was predictive in a relatively unfamiliar dyadic interaction (i.e., ‘taking’). Finally, in an unfamiliar and novel prize-preference task that did not include social interaction, both the BBB and the parental reports predicted children’s decisions. However, the BBB had an advantage in classifying children from both sides of the spectrum, i.e., high BIS-oriented children and high BAS-oriented (Fig 4B).

Based on the idea that approach-avoid conflicts are salient, particularly in ambiguous, or unfamiliar contexts [2, 6], we can understand *why* the BBB was predictive in some contexts and not others. Moreover, alongside the validation of the BBB, we may also discover *which* contexts may be perceived as ‘novel’ to children (e.g., in our case, monopolizing behavior for boys and prize choices for both genders). It is reasonable to assume that there is less ambiguity about how to behave in the presence of normative guidelines (e.g., social norms). In that case, an approach-avoid conflict would be weaker than in unfamiliar situations, wherein normative guidelines do not exist, thus allowing one’s BIS-BAS orientations to manifest predictably.

Integrating the domains of individual differences and decision-making also provides new results regarding ‘taking aversion’ and risky choice preferences. First, regarding ‘taking aversion’, research on children is scarce [58]; yet, a related phenomenon could be what is known as ‘advantageous inequity aversion’, suggesting that when experimenters set a distributive offer that favors one child compared to another, then 4- to 8-year-olds, across cultures, accept such self-advantageous offers [73]. Here, when children had to **initiate** a ‘taking’ behavior themselves (instead of accepting offers made by others), the inclusion of motivational orientation exposed those who are willing to cause advantageous inequity, i.e., BAS-oriented boys. Notably, we had no prior hypothesis regarding gender: On the one hand, inhibitory capabilities emerge earlier among girls [74], and thus may regulate the manifestation of their approach-avoidance motivations [2]. On the other hand, gender differences in ‘taking aversion’ were recently found among adults [56], when inhibitory capabilities have already been matured, thus calling for dedicated examination regarding the individual, normative, and contextual factors that may underlie ‘taking aversion’.

Second, regarding risky choice preferences in non-social scenarios, two sub-groups with opposite tendencies were discovered—one who strongly preferred to take home an ambiguous and ‘risky’ reward (i.e., unknown prize) and a second who strongly desired to take a ‘safe’ one (visible prize). Children’s prize preferences in our ecological task resonate with individual differences in novelty-seeking [75–77], which facilitate exploration [78], language learning [75, 76, 79], and pragmatic social skills [80, 81]—all of which arguably stem from differences in temperament and are also associated with maladaptive behavior in adulthood.

Some limitations should be noted. First, in terms of the structure of the BBB, our findings suggest that scores obtained via the BBB did not correlate with those of the caregiver’s reports. Previous studies that studied infants or early adolescents have also recorded discrepancies with parental reports [13, 25, 65]. To illustrate, one of the earliest studies [25] recruited 117 2-year-olds who received scores from their parents in being of either extremely inhibited or uninhibited temperament, yet after detailed behavioral and emotional coding, about 40% of them were characterized as neither inhibited nor uninhibited. Similar discrepancies were found also between the original scales and a simplified version for 8-12-year-olds [13]. Here, the adult questionnaire differs from the BBB in additional respects, such as the *number* of questions (24 compared to 12 items; respectively), differences in who the questions *refer to* (e.g., one’s own behavior or someone else’s behavior, respectively), and differences in the *type* of questions (e.g., ‘abstract’ compared to ‘concrete’, respectively) (see S1 Table in S1 File). To give one example, a relatively abstract item in the adult questionnaire such as “*I will often do things for no other reason than that they might be fun*”, was presented in the BBB with Vignettes showing a protagonist thinking about whether to walk around a puddle of water and remain dry or—jump inside just for fun. In other words, abstract questions could be taxing for some children, but at the same time, concrete questions could be too narrow to fit all children. Notwithstanding these differences, our findings do suggest that the BBB was highly predictive of children’s actual behaviors, which implies it may be viable for direct assessment of behavioral orientation at an early stage in development.

A second potential limitation could be methodological. Children were tested with the BBB first, then participated in one of two distributive tasks, and lastly chose a prize to take home. The rationale for starting with the BBB was to examine children without the concern of any carry-over effects which might result from the dictator games if those would have played first. The rationale for ending with the prize choice task was to keep it ecologically valid (i.e., children are rewarded at the end) and give the children the impression that the “experiment” was over. Nonetheless, using the BBB before, during, and after tasks, in a controlled manner, as well as at different time intervals, could be of great value, as some research has demonstrated the malleability of BIS-BAS orientation, across different developmental periods (e.g. between 3 to 9-years of age) [25, 26].

Overall, by systematically examining three contexts that vary in familiarity and sociality, the current study offers initial evidence for the ability to examine individual differences in decision-making via short-term behavioral tasks. Research on individual differences should benefit from a new, theoretically driven, direct measurement suited for early childhood, providing immediate added value. For instance, as “motivational imbalance” in early childhood correlates with maladaptive behavior in the social sphere [2, 16], educational policies and therapeutic programs can harness the BBB to identify and tailor child-specific interventions for social resilience and academic success. Even more broadly, knowing *who* the acting agent is (e.g., gender, age), *what* is the motivational orientation for action (e.g., approach, avoidance), and *what* is the type of context (e.g., familiar or not, ambiguous or not, social or not), may allow portraying a detailed, and theoretically coherent, description of the interplay between motivations, cognition, and behavior.

## Supporting information

S1 File Supplementary material includes a summary table of all items (S1 Table), specific coding scheme (S2-S6 Figs), additional analysis, and the set of pre-edited videos of various peers (S7 Fig). (DOCX)
