# Supplementary material for: Approach-avoidance orientations can predict young children’s decision-making
Source: PLoS One. 2023 Jul 24;18(7):e0288799. doi: 10.1371/journal.pone.0288799 (PMC10365306; doi:10.1371/journal.pone.0288799)
Supplement: S1 File — (DOCX) [file pone.0288799.s001.docx]

**Approach-avoidance orientations can predict young children’s decision-making.**

**Supplementary Material**

1. Bis-Bas Book
   1. The original BIS-BAS scales (Carver & White, 1994) consists of 24 items that represent four factors, i.e., BIS factor (7 items), BAS-Drive (5), BAS-Reward Sensitivity (4), BAS-Fun Seeking (4), and fillers (4). To address young children, we have simplified the original scales to create the BIS-BAS-Book. We created 12 pictorial items and maintained a similar ratio between subfactors, that is, 3 BIS items, 2 BAS-Drive, 2 BAS-Reward-sensitivity, 2 BAS fun-seeking, and three fillers. Original vignettes were created to correspond to the original ones. The protagonists were matched to the participant’s physical characteristics (i.e., gender and hair color) and personal ones (i.e., age and name, as stated by the Experimenter). The order of the items was randomized, and each provided a score between 1 and 4. Items are listed in Table-1S, and more detailed content and coding follow.

| Item# | Subscale | Original Item (Carver & White, 1994) | Vignette summary (the BIS-BAS-Book) |
| --- | --- | --- | --- |
| 1 | BIS | *Criticism or scolding hurts me quite a bit* | A protagonist in a messy room facing an angry parent |
| 2 | BIS | *I feel worried when I think I have done poorly at something important* | Several peers have completed a task successfully except the protagonist, who is lagging |
| 3 | BIS | *I worry about making mistakes* | A protagonist is facing a high Lego tower that may collapse |
| 4 | BAS-Drive | *I go out of my way to get things I want* | A protagonist has to choose between an OK reward which is easy to obtain and a much better reward that requires effort |
| 5 | BAS-Drive | *When I go after something, I use a “no holds barred” approach* | A protagonist has to choose between violating a norm to fulfill an immediate desire or restraining such a desire and complying with the norm |
| 6 | BAS-Reward sensitivity | *When I am doing well at something, I love to keep at it* | A protagonist is doing a great job (e.g., placing stickers in all the right places) and needs to decide whether to complete the task or quit doing something else. |
| 7 | BAS-Reward sensitivity | *When I get something I want, I feel excited and energized* | A protagonist obtains a highly desired reward (i.e., cookie jar) and wonders whether to eat a cookie or leave the jar and go outside to do something else |
| 8 | BAS – Fun-seeking | *I will often do things for no other reason than that they might be fun* | A protagonist sees a small puddle of water and wonders whether to walk around it (and remain dry) or jump inside just for fun. |
| 9 | BAS – Fun-seeking | *I often act on the spur of the moment* | A protagonist is walking and suddenly hears an unusual sound coming from behind. The question is whether to stop and check the source of the unusual sound or ignore it |
| 10 | Filler | A person’s family is the most important thing in life | A protagonist is playing with her parents and asked how it feels |
| 11 | Filler | It is hard for me to find the time to do things such as get a haircut | A protagonist is feeding ducks and asked how it feels |
| 12 | Filler | How I dress is important to me | A protagonist is wearing a new shirt in front of the mirror |

**S1 Table.** **A summary of the vignettes of the BBB.**

- 1. **Correlations of the BBB and Parental reports:** Looking at the BBB scores, two of three BAS sub-scales were significantly correlated, that is, *drive* and *fun-seeking* (r=.36; p<.005; one-tailed) and *fun-seeking* and *reward sensitivity* (r=.2; p<.025). Looking at the caregiver’s report, all three BAS sub-scales were correlated, that is, *drive* and *fun-seeking* (r=.63; p<.005), *fun-seeking* and *reward sensitivity* (r=.45; p<.005) and *reward sensitivity* and *drive* (r=.45, p<.005).
  2. **Detailed content and coding scheme for each item is as follows:**

- **Three BIS items**


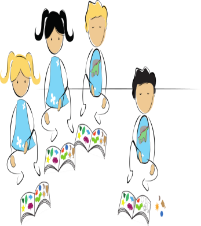

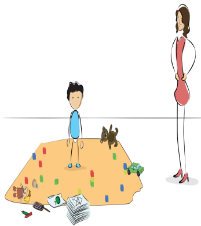

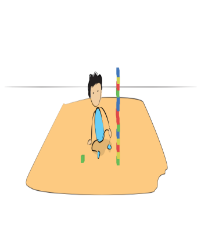


**1.**

**2.**

**3.**

**S2 Fig. Three BIS items**

1. Represents “Criticism or scolding hurts me quite a bit” (Item #8 in the original BIS-BAS scales).

Content: Scenario 1 portray a protagonist in a messy room facing an angry parent. The question is how does the protagonist feel when facing an angry parent (happy/ sad), and a follow-up question is “*Does it often happen that* {participant’s name} *feels this way? or only sometimes?*”

Coding: For answering that the protagonist feels sad and that such a feeling happens **often** or **sometimes**, the participant received a score of “1” or “2”, respectively (highest BIS scores). Following this logic, for answering that the protagonist feels happy and that such a feeling happens **sometimes** or **often** received a score of “3” or “4”, respectively (lowest BIS scores).

1. Represents “I feel worried when I think I have done poorly at something important” (Item #19 in the original BIS-BAS scales).

Content: Scenario 2 portrays children placing stickers in their sticker books. All have completed the task successfully except the protagonist, who is lagging. The question is whether the protagonist should stop placing the stickers or continue. A follow-up question is, “*Does it often happen that* {participant’s name} *acts this way? or only sometimes?*”

Coding: Similar to the logic of item 1 above, for answering that the protagonist should quit the task and that such behavior happens **often** or **sometimes**, the participant received a score of “1” or “2”, respectively (highest BIS scores). For answering that the protagonist should continue the task and that such behavior happens **sometimes** or **often** received a score of “3” or “4”, respectively (lowest BIS scores).

1. Represents “I worry about making mistakes” (Item #24 in the original BIS-BAS scales).

Content: Described in the main text (Figure 1).

Coding: Answering that the protagonist should not use the last block and that this behavior happens **often** receive a score of “1” (i.e., worrying about mistakes is “very true for me”). Children who answer that the protagonist should not use the last block and that this behavior happens **sometimes** receive a score of “2” (i.e., worrying about mistakes is “somewhat true for me”). Following this logic, children who answer that the protagonist should use the last block and state that such behavior happens **sometimes** or **often** receive a score of “3” or “4” (respectively).

**Notes**: We chose these items from the original scale to avoid redundancy:

- Chosen Item #8 is similar to (unchosen) Item #13:
- “I feel pretty worried or upset when I think or know somebody is angry at me.”
- Chosen item #24 is similar to (unchosen) items #16 and #22
- “If I think something unpleasant is going to happen I usually get pretty "worked up”.
- “I have very few fears compared to my friends.”
- **Two BAS-Drive items**


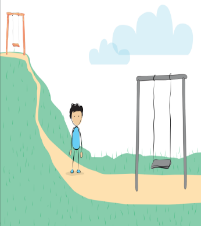

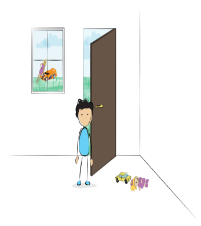

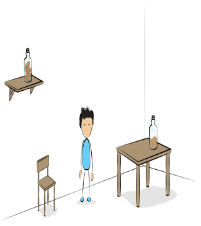


**4.a.**

**4.b.**

**4.c.**


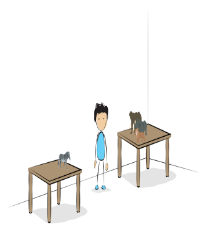

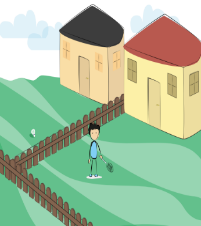

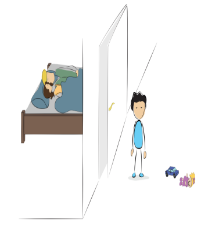


**5.a.**

**5.b.**

**5.c.**

**S3 Fig. Two BAS-Drive items**

1. Represents “I go out of my way to get things I want” (item #3 in the original BIS-BAS scales).

Content: Scenarios 4.a., 4.b., and 4.c., portray a choice between an “OK” reward that is easy to obtain (i.e., nearby cookies, toys, and a swing; respectively) OR – a “better” reward that requires effort to obtain (i.e., climbing a chair to get “great” cookies, going outside to get “better” toys, or climb a hill to try an “exciting” swing).

Coding: The child received a score of “1” for three conservative answers (i.e., picking the “OK” rewards, lowest Drive score), a score of “2” for two conservative answers, a score of “3” for one conservative answers, and a score of “4” for 0 conservative answers (highest Drive score).

1. Represents “When I go after something, I use a “no holds barred” approach” (item #21 in the original BIS-BAS scales).

Content: Scenarios 5.a., 5.b., and 5.c. portray a protagonist who has to choose between violating a norm to fulfill an immediate desire or restraining one’s immediate desire and complying with the norm. Specifically, item 5.a. describes a protagonist that wants to play with new noisy toys, but his parents are currently napping. The dilemma is between playing with noisy toys and risking waking up the parents OR waiting until the parents are awake and only then playing with the noisy toys.

Item 5.b. describes a protagonist that accidentally threw a ball over the neighbor’s fence and wanted to get it back and play. The dilemma is between climbing over the neighbor’s fence without permission to return the ball OR waiting for the neighbor to return home, asking for permission to get the ball, and only then playing. The specific text is detailed in the MS.

Item 5.c. describes a protagonist playing with toy animals and wishing to complete the Zoo with a nearby Zebra. The dilemma is between taking a nearby zebra that belongs to another child without permission and completing the Zoo, OR – waiting for the other child to come back, asking to borrow the zebra, and completing the “zoo”.

Coding: The child received a score of “1” for three conservative answers (i.e., norm compliance, lowest Drive score), “2” for two conservative answers, “3” for one conservative answer, and “4” for 0 conservative answers (highest Drive score).

**Notes**: We chose these items from the original scale to avoid redundancy:

- Chosen Item #3 is similar to (unchosen) Item #12:
- “If I see a chance to get something I want I move on it right away”.
- Chosen item #21 is similar to (unchosen) item #9
- “When I want something I usually go all-out to get it.”.
- **Two BAS-Reward sensitivity items**


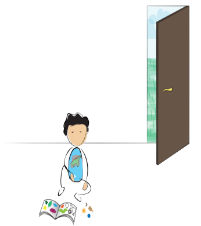

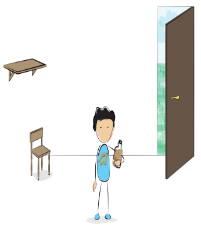


**6.**

**7.**

**S4 Fig. Two BAS-Reward sensitivity items**

1. Represents “When I’m doing well at something I love to keep at it” (item #4 in the original BIS-BAS scales)

Content: Scenario 6 portrays a protagonist who is doing a great job placing stickers in all the right places in the sticker book. There are few stickers left, and the question is whether the protagonist should keep completing the sticker book OR – go outside to do something else. Specific text is detailed in the MS.

Coding: For answering that the protagonist should quit and go outside and that such behavior happens **often** or **sometimes**, the participant received a score of “1” or “2”, respectively (lowest Reward sensitivity scores). Following this logic, for answering that the protagonist should complete the sticker book and that such behavior happens **sometimes** or **often** received a score of “3” or “4”, respectively (highest Reward sensitivity scores).

1. Represents “When I get something I want, I feel excited and energized” (item #7 in the original BIS-BAS scales)

Content: Scenario 7 portrays a protagonist who obtained a highly desired cookie jar and wondering whether to open it and eat a cookie OR – leave the jar behind and go outside to do something else.

Coding: A core of “1” was given for answering that the protagonist should leave the cookie jar behind and go outside (lowest Reward sensitivity score). Scores of “2”, “3”, or “4” were given for answering that the protagonist should eat a cookie and stating in a follow-up question that the cookie tastes **“OK”**, **“good”**, or **“awesome”**, respectively (highest Reward sensitivity scores).

**Notes**: We chose these items from the original scale to avoid redundancy:

- Chosen Item #4 is uniquely about ‘persistence’
- Chosen item #7 is similar to (unchosen) items #14, #18, and #23 (about excitatory arousal)
- “When I see an opportunity for something I like I get excited right away”.
- “When good things happen to me, it affects me strongly.”.
- “It would excite me to win a contest.”.
- **Two BAS-Fun-seeking items**


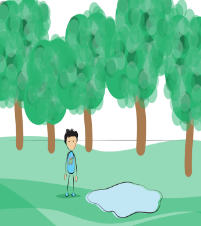


**8.**


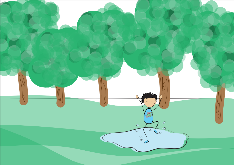

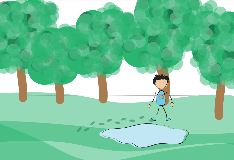

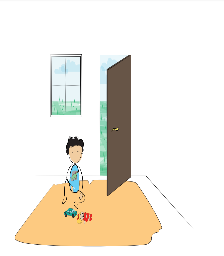

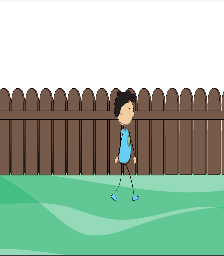

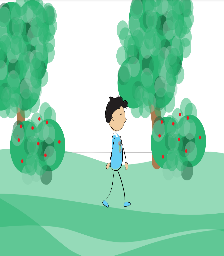


**9.a.**

**9.c.**

**9.b.**

?

?

?

**S5 Fig.** **Two BAS-Fun-seeking items**

1. Represents “I will often do things for no other reason than that they might be fun” (item #10 in the original BIS-BAS scales)

Content: Scenario 8 portrays a protagonist walking in the forest and seeing a small puddle of water. The question is whether the protagonist should walk around the puddle and remains dry OR – jump inside just for fun. Upon the participant’s answer, a follow-up question is asked: “*Does it often happen that* {participant’s name} *acts this way? or only sometimes?*”. The specific text is detailed in the MS.

Coding: For answering that the protagonist should walk around the puddle and remain dry, and answering that this behavior happens **often** or **sometimes**, the participant received a score of “1” or “2”, respectively (low Fun-seeking scores). Following this logic, for answering that the protagonist should jump into the puddle just for fun and that such behavior happens **sometimes** or **often**, the participant received a score of “3” or “4”, respectively (high Fun-seeking scores).

1. Represents “I often act on the spur of the moment” (item #15 in the original BIS-BAS scales)

Content: Scenario 9 portrays a protagonist who is walking/playing and suddenly hears an unusual sound coming from behind (e.g., “Boing!”). The question is whether the protagonist should stop his current activity and check the source of the unusual sound OR – ignore it.

Coding: The child received a score of “1” for three conservative answers (i.e., ignoring the strange sound, lowest Fun-seeking score), “2” for two conservative answers, “3” for one conservative answer, and “4” for 0 conservative answers (i.e., go and check the unusual sound, highest Fun-seeking score).

**Notes**: We chose these items from the original scale to avoid redundancy:

- Chosen Item #10 is similar to (unchosen) item #5
- “I'm always willing to try something new if I think it will be fun.”.
- Chosen item #15 is similar to (unchosen) item #20
- “I crave excitement and new sensations.”.
- **Three Filler items**


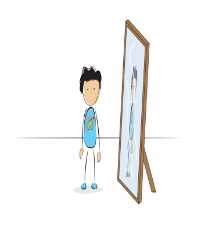

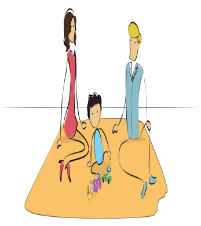

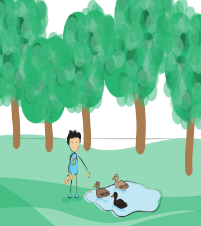


**10.**

**11.**

**12.**

**S6 Fig.** **Three filler items.**

1. Represents “A person’s family is the most important thing in life” (item #1 in the original BIS-BAS scales)

Content: Scenario 10 portrays a protagonist playing with his/her parents. The question is how does the protagonist feel when doing so (i.e., happy/ sad), and a follow-up question is “*Does it often happen that* {participant’s name} *feels this way? or only sometimes?*”

Coding: Irrelevant.

1. Represents “It is hard for me to find the time to do things such as to get a haircut” (item #1 in the original BIS-BAS scales)

Content: Scenario 11 portrayed a protagonist who is feeding ducks. The question is how does the protagonist feel when doing so (i.e., happy/ sad), and a follow-up question is “*Does it often happen that* {participant’s name} *feels this way? or only sometimes?*”

Coding: Irrelevant.

1. Represents “How I dress is important to me” (item #6 in the original BIS-BAS scales)

Content: Scenario 12 portrays a protagonist wearing a new shirt and looking at the mirror. The question is how does the protagonist feel when doing so (i.e., happy/ sad), and a follow-up question is “*Does it often happen that* {participant’s name} *feels this way? or only sometimes?*”

Coding: Irrelevant.

1. Dictator-game phase (‘giving’ and ‘taking’)

For the ‘giving’ and ‘taking’ social conditions, participants played with an unfamiliar, gender- and age-matched peer. Each participant played with only one peer. To exclude the possibility of bias towards particular attributes of a peer, we filmed sixteen different children in advance, forming a set of 4-6-year-olds and 6-10-year-olds. Each participant interacted with a random peer similar in gender and age.

**S7 Fig.** **Set of pre-edited videos of various peers.** Comprised of relatively young children (i.e., 4-6-years-of-age) **(A)** and older ones (6-10-years-of-age) **(B)**


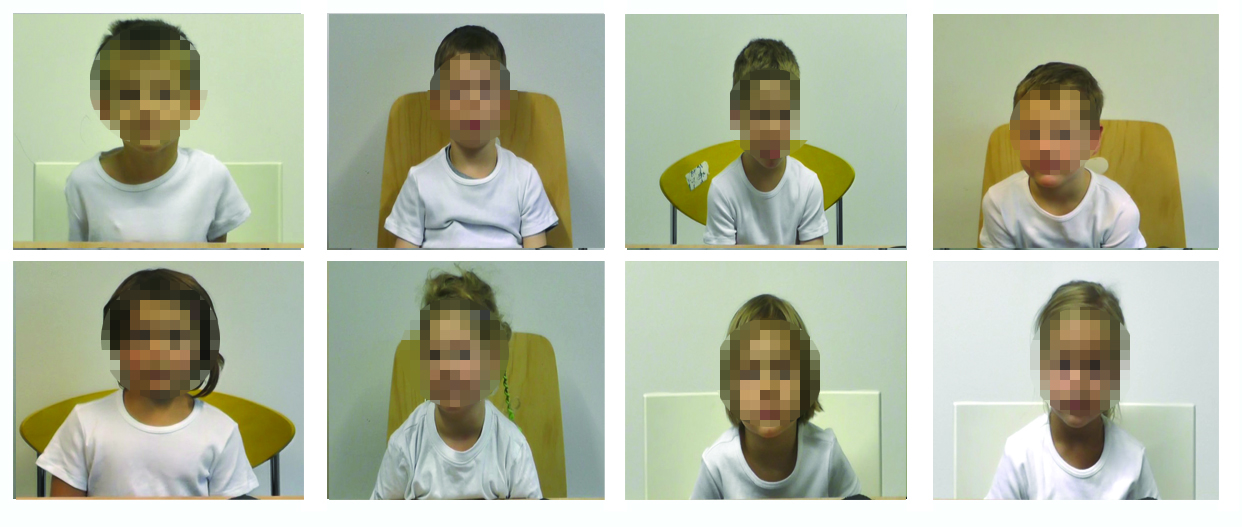


**A.**


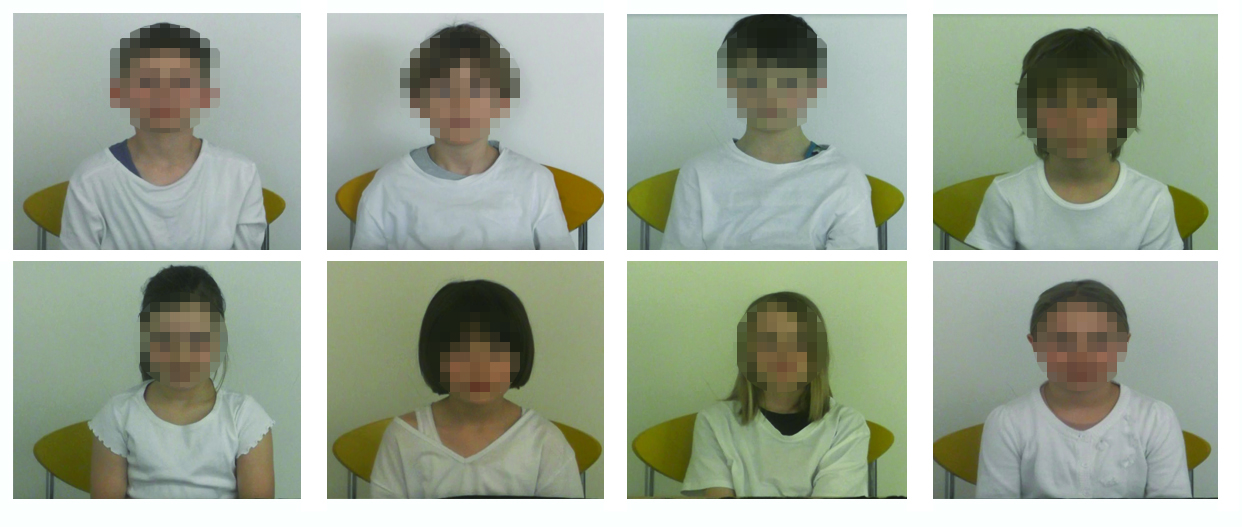


**B.**
